# Hydroxycinnamyl Derived BODIPY as a Lipophilic Fluorescence Probe for Peroxyl Radicals

**DOI:** 10.3390/antiox9010088

**Published:** 2020-01-20

**Authors:** Jaroslaw Kusio, Kaja Sitkowska, Adrian Konopko, Grzegorz Litwinienko

**Affiliations:** 1 Faculty of Chemistry, University of Warsaw, Pasteura 1, 02-093 Warsaw, Poland; j.kusio@chem.uw.edu.pl (J.K.); akonopko@chem.uw.edu.pl (A.K.); 2 Centre for Systems Chemistry, Stratingh Institute for Chemistry, University of Groningen, Nijenborgh 4, 9747 AG Groningen, The Netherlands; 3 Nencki Institute of Experimental Biology, Polish Academy of Sciences, 3 Pasteur St, 02-093 Warsaw, Poland

**Keywords:** antioxidant activity, peroxyl radicals, fluorescent probes, rate constant, micelles

## Abstract

Herein, we describe the synthesis of a fluorescent probe **NB-2** and its use for the detection of peroxyl radicals. This probe is composed of two receptor segments (4-hydroxycinnamyl moieties) sensitive towards peroxyl radicals that are conjugated with a fluorescent reporter, dipyrrometheneboron difluoride (BODIPY), whose emission changes depend on the oxidation state of the receptors. The measurement of the rate of peroxidation of methyl linoleate in a micellar system in the presence of 1.0 µM **NB-2** confirmed its ability to trap lipid peroxyl radicals with the rate constant *k*_inh_ = 1000 M^−1^·s^−1^, which is ten-fold smaller than for pentamethylchromanol (an analog of α-tocopherol). The reaction of **NB-2** with peroxyl radicals was further studied via fluorescence measurements in methanol, with α,α′-azobisisobutyronitrile (AIBN) used as a source of radicals generated by photolysis or thermolysis, and in the micellar system at pH 7.4, with 2,2′-azobis(2-amidinopropane) (ABAP) used as a thermal source of the radicals. The reaction of **NB-2** receptors with peroxyl radicals manifests itself by the strong increase of a fluorescence with a maximum at 612–616 nm, with a 14-fold enhancement of emission in methanol and a 4-fold enhancement in the micelles, as compared to the unoxidized probe. Our preliminary results indicate that **NB-2** behaves as a “switch on” fluorescent probe that is suitable for sensing peroxyl radicals in an organic lipid environment and in bi-phasic dispersed lipid systems.

## 1. Introduction

**Scheme 1:**
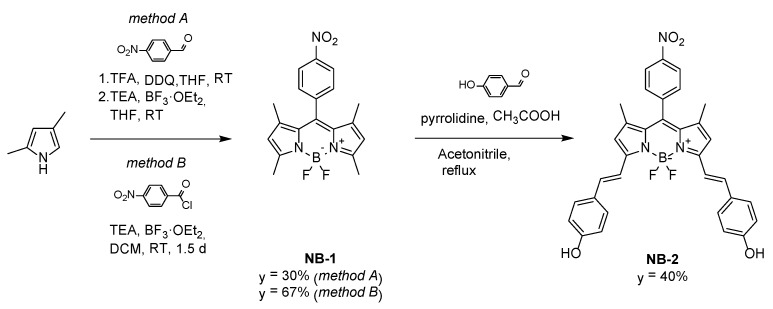
Methods A and B for the synthesis of BODIPY derivative with nitrophenyl at mezo-position (**NB-1**) and a subsequent attachment of two hydroxycinnamyl residues at positions 3 and 5 leading to derivative **NB-2** (TFA = trifluoroacetic acid, DDQ = 2,3-dichloro-5,6-dicyano-1,4-benzoquinone, TEA = triethylamine, THF = tetrahydrofuran).

There is much evidence of the harmful effects of excess amounts of Reactive Oxygen Species (ROS) resulting in oxidative stress, and consequently, in undesired effects for health [1,2,3,4,5,6,7,8]. Excessive generation of ROS correlates with mitochondrial dysfunction and increased receptor signaling in cells in general [9]. Lipids are the main components of biomembranes and their exposition to endogeneous or exogeneous ROS initiates a peroxidation affecting the assembly, composition, polarity, structure, and dynamics of cellular membranes, and finally, can lead to the death of the cell [10]. Once the peroxidation is triggered by radical forms of ROS attacking the lipid (LH, Reaction (1) in Scheme 1), the process is propagated by alkyl and alkylperoxyl radicals (L• and LOO•, respectively) and in cyclic reactions of H abstraction (Reaction (2)) and O_2_ addition (Reaction (3)) tens and hundreds of lipid molecules are converted into the hydroperoxides until the process is terminated in Reaction (4).

*initiation:*      ROS + LH → L•             ; *R*_i_ (1)

*propagation:*      L• + O_2_ → LOO•           ; *k*_2_ (2)

       LOO• + LH → LOOH + L•           ; *k*_p_ (3)

Termination*:* Radicals (L•/LO•/LOO•) → non radical product   ; *k*_t_ (4)

where *R*_i_ is the rate of initiation and *k*_2_, *k*_p_*,* *k*_t_ are the rate constants for Reactions (2)–(4).

A number of problems connected with the oxidative stress and with mechanism of action still remains unsolved. For example, some compounds designed to eliminate ROS, which positively passed tests in vitro, show dramatically low activity in vivo [11]; therefore, a deeper insight is needed into the localization of ROS and their interactions with other components of living cells. Cellular concentrations of ROS are usually at the pico to nanomolar level and can change rapidly [12], thus, the methods used for their monitoring should be fast, efficient, and highly sensitive. Additionally, a molecular marker for oxidative stress should neither affect the process to be monitored nor the components of the cell.

Among the main methods of ROS detection the Electron Paramagnetic Resonanse EPR a technique using spin traps (usually nitroxides or nitrones) can be conveniently applied to living organisms. However, the expensive equipment and advanced methodology of the interpretation of the results make EPR rather inaccessible for non-specialists. Another method, Magnetic Resonance Imaging (MRI) [13,14], is often too expensive and time consuming to be broadly used for monitoring the oxidative stress on the cellular level (in vitro and in vivo). ROS can also be monitored by fluorescence imaging [12,15,16], a technique employing molecules of a fluorescence probe (FP) that allows for the observation of processes in single cells or their parts (via confocal microscopy) [17]. FP should react with ROS faster than other biomolecules and such a reaction should result in a clear fluorescence response (on/off), even at low concentrations of ROS.

**Figure 1:**
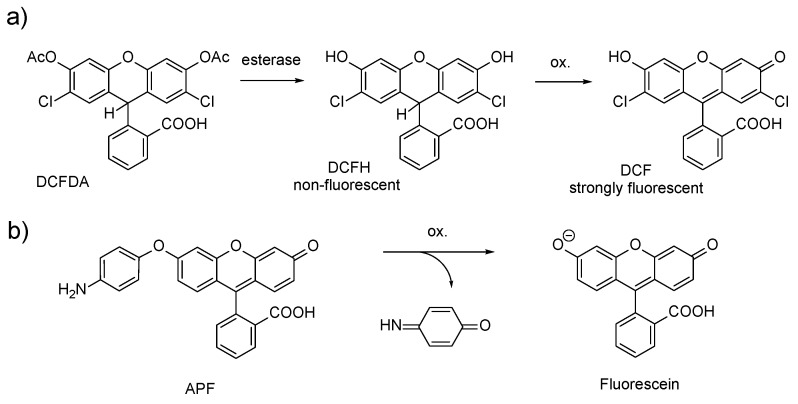
Mode of action of commonly used Reactive Oxygen Species (ROS) probes: (**a**) DCFH, (**b**) APF. 2,7-dichlorodihydrofluorescein (DCFH), and 2-[6-(4′-amino)phenoxy-3H-xanthen-3-on-9-yl] benzoic acid (APF).

**Figure 2:**
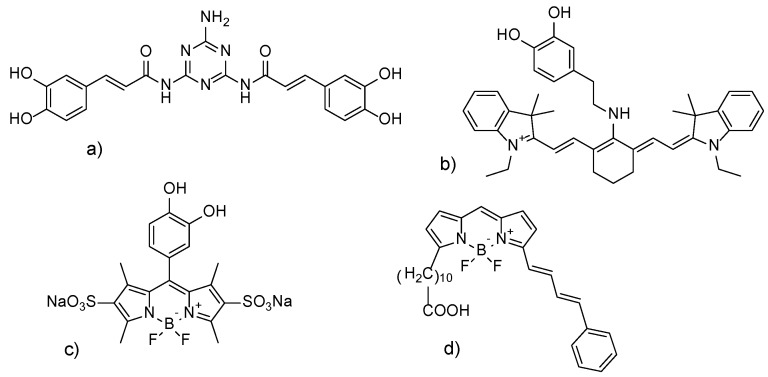
Structures of ROS fluorescent probes of receptor-reporter type with catechol moiety (**a**–**c**) and with conjugated diene interconnection (**d**) used as receptor.

Figure 1 presents examples of two FPs commonly used for the detection of intracellular ROS [18,19]. The diacetate form of dichlorodihydrofluorescein (DCFDA) after crossing the cell membrane is enzymatically hydrolyzed to polar form (DCFH, non-fluorescent), reacts (non-selectively) with ROS, and forms fluorescent DCF. Another commonly used FP, APF, is less sensitive, but more selective than DCFH. It reacts with **^•^**OH, HOCl, and ONOO**^-^**. DCFH and APF are the “switch on” fluorescent probes, their reaction with ROS result in fluorescent products. This is, however, not the only mechanism FPs can follow. Some FPs can act as “switch off” markers, i.e., their fluorescence is quenched after the reaction with ROS. In both cases, the fluorescence signal/intensity (on/off) of the whole molecule strictly depends on the reaction with ROS. In the first case, an electron is promoted from the ground state to the excited singlet state of the molecule: 1GS → 1ES* and the reaction of 1ES* with ROS results in a relaxation to a lower energy triplet state: 1ES* → 3ES*. Relaxation of this state can be either mediated again by triplet oxygen or can happen via a heat dissipation route. The second mechanism involves photoinduced electron transfer (PeT), that is, LUMO → LUMO transfer within the excited donor–acceptor pair [D*:A] → [D+:A−]*, then [D+:A−]* returns to the ground state either with or without photon emission (exciplex emission) with the subsequent return of electron to the donor (HOMO → HOMO) and decomposition of the D:A complex. The same PeT mechanism might operate for D and A covalently bonded as two structurally and functionally different segments. The first segment is responsible for the reaction with ROS and is called a receptor (R), while the second one is called a reporter (F) and is a fluorophore whose fluorescent properties will change depending on the redox status of R. Recently, Tang and coworkers designed a “switch on” type of FP with a tripolycyanamide scaffold as F and two catechol moieties as receptors for O_2_^•−^ (Figure 2a) [20].

An example of a “switch off” catechol based sensor used for the detection of **^•^**OH and H_2_O_2_ is presented in Figure 2b [21]. The catechol functionality was also utilized in a dipyrrometheneboron difluoride (BODIPY) based fluorescent probe, shown in Figure 2c. This probe, however, is sensitive towards hypochlorous acid and hypochlorite [22]. Another probe, a lipophilic derivative of BODIPY (Figure 2d), with sensitivity to oxidation comparable to that of endogenous fatty acyl moieties, was proposed by Drummen and coworkers as FP for monitoring lipid peroxidation and antioxidant efficacy [23].

**Figure 3:**
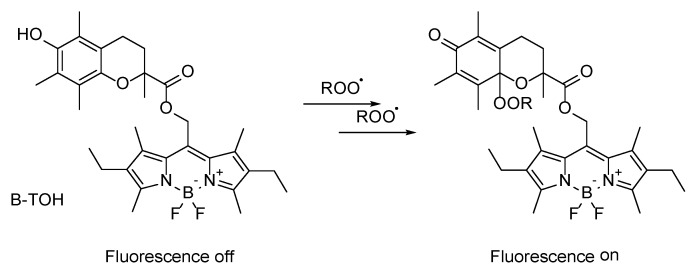
Mode of action BODIPY-tocopherol (B-TOH) peroxide fluorescent probe invented by Cosa [24].

Recently, Cosa and his group designed a series of BODIPY-α-tocopherol (and other phenolic antioxidants) FPs with excellent sensitivity towards trace amounts of peroxyl and alkoxyl radicals (“switch on” probes, see Figure 3) [24,25,26,27,28].

Herein, we describe the synthesis and preliminary results obtained for a molecule in which the reporter moiety (BODIPY) is covalently bonded to two receptor segments, sensitive toward peroxyl radicals, i.e., two phenol moieties connected via double C=C bonds to position 3 and 5 of the BODIPY core (Scheme 1).

Phenolic segments in **NB-2** are responsible for scavenging peroxyl radicals. In this particular case we deliberately used simple phenol moieties (monohydroxyl, non-hindered phenols) that are less reactive towards peroxyls than other natural antioxidants (tocopherols, carotenoids, and melatonin). Such a choice can be rationalized because our FP was designed for monitoring the system at the moment when other (more reactive) antioxidants have been consumed. The nitrophenyl moiety present at the meso-position of BODIPY is an example of a functional group that can be relatively easily converted into other functionalities in order to assemble the FP with other molecules or nanoparticles via the aromatic ring.

## 2. Materials and Methods

### 2.1. Chemicals and Reagents

Chemicals and reagents for synthesis: starting materials, reagents, and solvents were purchased from Sigma–Aldrich, Acros Organics and Combi-Blocks, and were used without any additional purification. The reaction progress was monitored by Thin Layer Chromatography performed on commercial Kieselgel 60, F254 silica gel plates with fluorescence-indicator UV254 (Merck, TLC silica gel 60 F254). For the detection of components, a UV light at λ = 254 nm or λ = 365 nm was used.

Chemicals and reagents for kinetic measurements: Initiator, α,α′-azobisisobutyronitrile, AIBN (>98% GC, Fluka Chemika), was recrystallized from methanol before use. 2,2’-Azobis(2-amidinopropane) dihydrochloride (ABAP; 97%), Triton X-100 (polyethylene glycol p-(1,1,3,3-tetramethylbutyl)-phenyl ether, 98%), methyl linoleate (LinMe; 99%), and 2,2,5,7,8-pentamethylchroman-6-ol (PMHC; ≥99%) were purchased from Sigma–Aldrich and were used as received.

### 2.2. General Information

^1^H, ^13^C, and ^19^F NMR spectra (in CDCl_3_ or *d_8_-*THF) were recorded at room temperature (25 °C) using Bruker Avance (300 or 500 MHz) or Agilent Technologies 400-MR (400/54 Premium Shielded) spectrometers (400 MHz) and edited using MestReNova 10.0 software. Chemical shifts for the specific NMR spectra were reported relative to the residual solvent signals in ppm; CDCl_3_: *δ*_H_ = 7.26; CDCl_3_: *δ*_C_ = 77.16; THF*-d_8_*: *δ*_H_ = 1.72, 3.58; and THF*-d_8_*: *δ*_C_ = 67.21, 25.31. HRMS ESI-MS spectra were acquired on a Micromass LCT ESI-TOF mass spectrometer equipped with an orthogonal electrospray ionization source. Column chromatography was performed on commercial Kieselgel 60, 0.04–0.063 mm, Fluka. Melting points were recorded using the OptiMelt Automated Melting Point System from Stanford Research Systems.

### 2.3. Synthesis Procedures

8-nitrophenyl-1,3,5,7-tetramethylpyrromethene fluoroborate (**NB-1**).

Method A (see Scheme 1): 2,4-dimethylpyrrole (0.50 mL, 457 mg, 4.8 mmol, 2.2 equiv.) and *p*-nitrobenzaldehyde (330 mg, 2.18 mmol) were dissolved in dry THF (45 mL) under a nitrogen atmosphere and the mixture was stirred for about 10 min. Then, 2 drops of trifluoroacetic acid were added and the reaction mixture was stirred at room temperature overnight. Next, 2,3-dichloro-5,6-dicyano-1,4-benzoquinone (500 mg, 2.2 mmol, 1 equiv.) in dry THF (40 mL) was added drop by drop. After stirring for 4–5 h the flask was opened and triethylamine (TEA, 3.0 mL, 2.18 g, 21.5 mmol, 9.7 equiv.) was added. The flask was closed and the reaction mixture was stirred for an additional hour. Subsequently, it was flushed with nitrogen and BF_3_·OEt_2_ (4.0 mL, 32.4 mmol, 14.8 equiv.) was added rapidly (either super-fast drop by drop or a steady stream). After the mixture was further stirred for 12 h, the solvent was evaporated and the crude reaction mixture was purified by column chromatography using pentane/diethyl ether 2/1 − 1/1 −> DCM to obtain the product as an orange-red solid (250 mg, 30% yield).

Method B (see Scheme 1): Under nitrogen atmosphere 2,4-dimethylpyrrole (0.610 mL, 5.93 mmol, 2.2 equiv.) was slowly added to a DCM (50 mL) solution of 4-nitrobenzoyl chloride (500 mg, 2.69 mmol). The reaction mixture was stirred overnight at room temperature. Next, the flask was opened and TEA (3.00 mL, 21.5 mmol, 8.0 equiv.) was added and the reaction mixture was stirred (still open) for an hour. Then, the flask was closed, flushed with N_2_, and BF_3_ etherate (4.00 mL, 32.4 mmol, 12 equiv.) was quickly added (fast drop by drop or a steady stream). After stirring the reaction mixture for the next hour, the solvents were evaporated and the crude mixture was purified by flash chromatography (dry loading) using pentane/diethyl ether (gradient 3/1 −> 2/1; *v/v*) as the eluent. Compound **NB-1** was obtained as red precipitate (670 mg, 67% yield). Both methods gave the same compound: ^1^H NMR (300 MHz, Chloroform-*d*) *δ* 1.39 (s, 6H), 2.59 (s, 6H), 6.04 (s, 2H), 7.57 (d, J = 8.8 Hz, 2H), 8.41 (d, J = 8.8 Hz, 2H), ^19^F NMR (282 MHz, Chloroform-*d*), and *δ* −146.24 (dd, J = 65.4, 32.6 Hz). LRMS (ESI+) calculated for [M + Na]^+^ (C_19_H_18_BF_2_N_3_O_2_Na): 392.2, found: 392.4. ^1^H spectrum was in agreement with published data [29].

8-nitrophenyl-3,5-bis(ethene-2,1-diyl)diphenol-1,7-dimethylpyrromethene fluoroborate (**NB-2**).

**NB-1** (50.0 mg, 0.135 mmol), 4-hydroxybenzaldehyde (10 equiv.), pyrrolidine (40 µL, 0.474 mmol, 3.5 equiv.) and acetic acid (38.7 µL, 0.677 mmol, 5 equiv.) and dry acetonitrile (6 mL) were placed in a round bottom flask and boiled under reflux until the color of the reaction mixture became green/blue. Subsequently, the reaction mixture was allowed to cool down and the solvent was evaporated. The crude product was then purified using flash chromatography using mixtures of DCM and diethyl ether or acetone as the eluents (pure DCM −> DCM/diethyl ether 85/15; *v/v*). Green solid, 32 mg, 40% yield, ^1^H NMR (400 MHz, THF-*d*_8_) *δ* 1.42 (s, 6H), 6.73 (s, 2H), 6.79 (dq, *J* = 8.8, 2.1 Hz, 4H), 7.31 (dd, *J* = 16.3, 3.0 Hz, 2H), 7.43 − 7.50 (m, 4H), 7.57 (d, *J* = 16.3 Hz, 2H), 7.63 − 7.74 (m, 2H), 8.40 (dq, *J* = 8.8, 2.1 Hz, 2H), 8.74 (brs, 2H). ^13^C NMR (101 MHz, THF-*d*_8_) *δ* 16.0, 117.5, 117.9, 119.5, 125.8, 130.1, 130.8, 132.4, 134.1, 136.7, 138.4, 142.4, 144.1, 150.3, 155.3, and 161.0. ^19^F NMR (376 MHz, THF-*d*_8_) *δ* −136.86 (dd, *J* = 67.0, 33.1 Hz). HRMS (ESI+) calc. for [M + Na]^+^ (C_33_H_26_BF_2_N_3_O_4_Na): 600.1888, found: 600.1874. Spectra ^1^H, ^13^C, ^19^F NMR and HRMS are presented in Supplementary Materials (Figures S1–S4).

### 2.4. Preparation of Micelles

The micelles were prepared using a methodology described in our previous kinetic studies [30,31]. Glass test-tubes with 19 µL of methyl linoleate (LinMe) and 10.5 mL of 16 mM Triton X-100 were stirred using Vortex for 60 sec. Then, 10.5 mL of buffer was added and the mixture was shaken again for 60 s. For UV-vis measurements, 2 mL of analogous samples were prepared, i.e., 2.1 µL of LinMe, 1.0 mL of 16 mM Triton X-100, and 1 mL of buffer solution were mixed. The final concentration of LinMe was always 2.73 mM. Buffer compositions: pH 4.0 acetate (82 mM CH_3_COOH; 18 mM CH_3_COONa), pH 7.0 Tris (50 mM 2-amino-2-(hydroxymethyl)propane-1,3-diol (TRIS), 50 mM HCl), and pH 7.4 phosphate (25 mM KH_2_PO_4_; 14.5 mM NaOH).

### 2.5. Methodology of Autoxidation Measurements

The ability of **NB-2** to trap peroxyl radicals was evaluated by monitoring the rate of peroxidation of methyl linoleate dispersed in the micellar system. The uptake of dissolved oxygen during peroxidation of micelles was carried out at 37 °C by using a RC650 Respirometer (Strathkelvin Instruments) equipped with a Clark-type electrode in the same way as described in one of our previous papers [30,31,32]. The samples were buffered at pH 7.0 with a Tris buffer and the chambers with magnetic stirring, containing 3 mL of micelles were saturated with oxygen. The electrode was placed inside the chambers and peroxidation was initiated by the injection of water solution of ABAP (final concentration 10 mM). After 10% of oxygen was consumed, 10 µL of the studied compound (PMHC, **NB-1**, **NB-2**) in ethanol was added. In this way, the final concentration of the added compounds was 1.0 µM).

### 2.6. UV-vis Studies of Stability and Reactivity in Methanol and in Micelles

UV-vis spectra of **NB-2** were recorded on Varian Cary 50 spectrometer (Agilent Technologies, Santa Cara, CA, USA) using quartz cuvettes QS with 10 mm optical path length. Stability measurements: the UV-vis spectra were recorded for 180 min for **NB-2** in methanol at 37 °C. For reactivity studies in methanol, peroxyl radicals were generated from AIBN by thermolysis (30, 37 and 50 °C, spectra were recorded every 5 min) or photolysis (ambient temperature).

For studies of reactivity with peroxyl radicals in micelles at pH 4.0 and 7.0, water soluble initiator (ABAP) was used: 130 µL **NB-2** in ethanol and 100 µL of aqueous solution of ABAP were injected to a quartz cuvette (with magnetic stirring), containing 2 mL of micelles. The final concentrations were 2.73 mM LinMe, 8 mM Triton X-100, 25 mM ABAP, 9.0 µM **NB-2**. UV-vis spectra were recorded every 15 min.

### 2.7. Spectrofluorometric Measurements

Spectrofluorometric measurements were performed on Cary Eclipse fluorescence spectrophotometer equipped with Peltier System and magnetic stirrer (Agilent Technologies, Santa Cara, CA, USA). Measurements were performed in the liquid phase (methanol or micellar system) using quartz cuvettes QS for fluorescence with 10 mm optical path length. The most frequently used configuration parameters were λ_ex_ = 575 nm, slit = 5 and 5 nm, and gain = medium.

Experiments in methanol: a stock solution of AIBN in methanol was added with a syringe into a thermostated quartz cell containing **NB-2** dissolved in methanol. The final concentrations were 15 or 10 μM of **NB-2** and 5 mM AIBN. For micellar systems, water soluble azo-initiator (ABAP) was used, but the employed methodology remained the same, and the samples contained 2.73 mM LinMe, 8 mM Triton X-100, 10 µM **NB-2**, and 10 mM ABAP. Automatic spectra collection was immediately started after the initiator was injected into the sample. The sample solution inside the cuvettes was non-stop stirred with a magnetic stirrer. Excitation and emission spectra were recorded at room temperature or at 30, 37, and 50 °C, depending on the experiment. Temperature, time of experiment, and intervals for spectra collection varied between experiments and are described for every measurement individually.

### 2.8. Photolysis of AIBN

In order to produce peroxyl radicals by the photolytic decomposition of AIBN, a solution of 15 μM **NB-2** in methanol was placed in a fluorescence quartz cell (optical path 10 mm) and methanolic solution of AIBN was injected into it with a glass microsyringe (final concentration of AIBN was 5 mM). The solution was continuously stirred with a magnetic bar and the cell was periodically irradiated for 5 s with 365 nm UV High Power LED irradiation (2.7 W and 1200 mW radiant power). After every 60 s, an emission spectrum was recorded and the process was repeated for the same sample. Measurements were conducted at room temperature. The photography of UV irradiation chamber is presented in Figure S5 (Supplementary Material). The same methodology was used for UV-vis measurements.

## 3. Results and Discussion

### 3.1. Synthesis and Spectral Chracteristics of **NB-2**

**NB-1** was prepared using two different methods. As the yield of the product obtained by method A was not satisfactory, we employed the conditions described in method B, see Scheme 1. With acyl chloride instead of aldehyde as the substrate, the synthesis of **NB-1** was much cleaner, and the compound could be easily isolated with a 67% overall yield. Then, Knoevenagel condensation was carried out in refluxing acetonitrile with freshly dried molecular sieves, as proposed by Cicchi and coworkers [33]. This method gave **NB-2** with a satisfactory yield of 40%. **NB-2** is soluble in alcohols (methanol, ethanol), acetonitrile, THF, ethyl acetate, and acetone. Moderate solubility is observed in CH_2_Cl_2_ and CHCl_3_. We did not measure the octanol/water partition coefficient, but we roughly assumed that **NB-2** should be similar or even more lipophilic than the fluorescent probe with carboxyl functionality presented in Figure 2d, for which Drummen et al. determined log*P* = 2.49 ± 0.04 at pH 7.4 (i.e., in its anionic form) [23].

**Figure 4:**
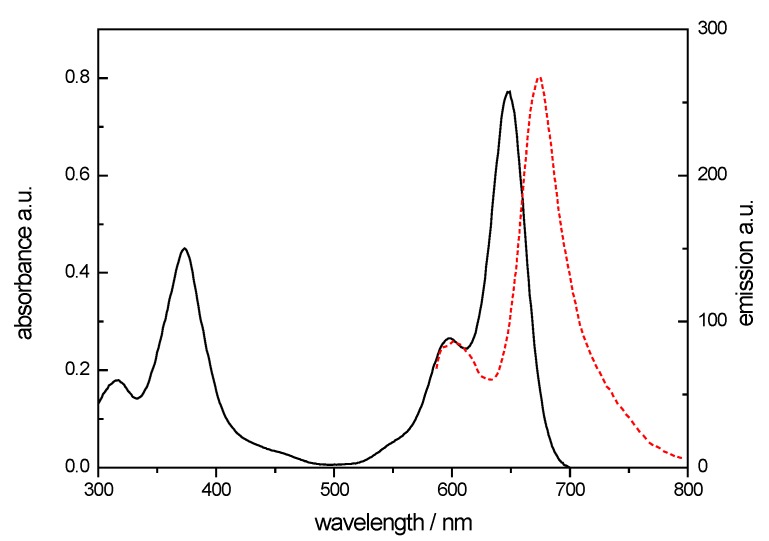
Absorbance and emission spectra of 15 μM **NB-2** in methanol at ambient temperature: Black solid line: UV-vis absorption spectrum. Red dashed line: emission spectrum at 585–800 nm (λ_excitation_ = 575 nm, emission and excitation slit = 5 nm, and detector gain = medium).

Figure 4 shows the absorption and emission spectra of **NB-2** in methanol at 23 °C. The compound had its absorption maximum at λ = 647–653 nm (molar absorptivity has been calculated as ε_649nm_ = 5.1 × 10^4^ M^−1^·cm^−1^) with a shoulder band with a local maximum at 596 nm, and strong absorption at 373 nm (ε_373nm_ = 3.0 × 10^4^ M^−1^·cm^−1^).

In most cases, boron dipyrromethene difluoride (BODIPY)-based dyes are fluorochromes that display bright green fluorescence in the range of 450 to 550 nm. **NB-2** is a derivative in which the boron dipyrromethene difluoride core is substituted with a nitrophenol at the meso-position, and two phenolic moieties via stilbene-like interconnections. Due to the extended conjugation, the intact **NB-2** displays a bright red fluorescence (see photography/Figure S5) with an emission maximum at 674 nm (red dashed line in Figure 4).

Stability of **NB-2** in aerated methanol at 37 °C was examined using UV-vis measurements (for details, see Figure S6). No changes in the absorbance of the compound were observed during at least 180 min. Considering the fact that most further measurements conducted in this study lasted max. 120 min in similar conditions, we can assume that all of the color and emission changes that occurred during tests came from the reaction with radicals.

### 3.2. Kinetic Parameters of Reaction of **NB-2** with Peroxyl Radicals

**Figure 5:**
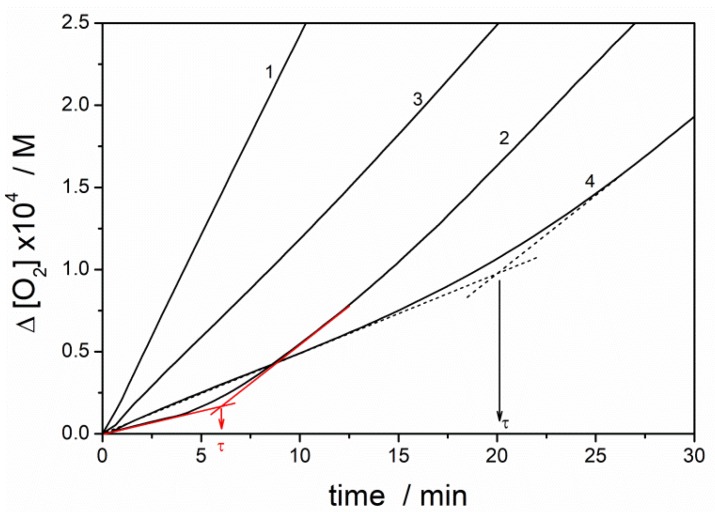
Oxygen uptake plots for autoxidation 8 mM Triton X-100 micelles containing 2.73 mM methyl linoleate initiated with 10 mM ABAP at 37 °C at pH 7.0. Spontaneous oxidation (1), oxidation in presence of 1.0 μM PMHC (curve 2), 1.0 μM **NB-1** (curve 3), and 1.0 μM **NB-2** (curve 4). The induction times (τ) for curves 2 and 4 are defined.

Two features are crucial for a molecule to be used as FP for sensing /imaging peroxyl radicals: (i) the molecule should exhibit relatively high reactivity toward alkylperoxyl radicals, and (ii) such a reaction should result in an increase or decrease of fluorescence (switch on/off FP). Therefore, in the first step, we examined the ability of **NB-2** to react with peroxyl radicals mediating lipid peroxidation in a micellar system. We selected methyl linoleate (LinMe) as a lipid representing polyunsaturated fatty acids (PUFA)—the constituents of natural lipid bilayers and biomembranes. During the measurement, thermal decomposition of water soluble azo-initiator ABAP produces primary radicals (R•) that are immediately converted into water soluble peroxyl radicals able to abstract the H atom from the weakest C-H bond (bis-allyl position) at the LinMe molecule. This process is regarded as the initiation of peroxidation (Reaction (1)) and triggers the propagation described by Reactions (2) and (3). The typical plot of oxygen consumption of such spontaneous PUFA peroxidation is presented in Figure 5 as curve 1, with the rate of oxidation *R*_ox_ = d*[O*_2_*]/*d*t*.

The rate of peroxidation can be significantly reduced in the presence of small amounts of chain-breaking antioxidants (CBA)—small molecules, mainly phenols, thiols, aromatic amines, or terpenoids that sacrificially eliminate peroxyl radicals from the propagation chain, Reaction (5) [34].

LOO• + CBA → LOOH + CBA_(−H•)_  ; *k*_inh_. (5)

Three features are important to be effective CBAs: first, their reaction with radicals (Reaction (5)) should be much faster than the propagation step of peroxidation (*k*_inh_ >> *k*_3_), secondly, the products of Reaction (5) should not be highly reactive radicals themselves, and finally, as the propagation occurs within a lipid membrane, an efficient CBA should be localized inside it. An example of such a compound is PMHC (2,2,5,7,8-pentamethylchroman-6-ol), an analogue of α-tocopherol (the most active natural CBA). The addition of 1.0 μM PMHC into the system causes a lag phase (induction period within time τ, see curve 2 in Figure 5). The observed time τ is dependent on the initial concentration of antioxidant, [ArOH]_0_, and the rate of initiation, *R*_i_:

τ = *n* [ArOH]_0_/*R*_i_. (6)

*R*_i_ can be determined from transformed Equation (6) when τ is measured for a known concentration of PMHC as a standard CBA, for which *n* = 2.0. The inhibition rate constant *k*_inh_ can be determined from an integral form of the rate equation:

(7) $$∆{\left[O_{2}\right]}_{t}=-\frac{k_{p}\;\left[\mathrm{LH}\right]}{k_{inh}}\ln\left(1-\frac{t}{\tau }\right),$$

where ∆[O_2_]_t_ stands for molar oxygen consumption [M] recorded at time t within the induction period (t < τ). With *k*_p_ = 36 M^−1^·s^−1^ for methyl linoleate dispersed in Triton X-100 micelles (assuming the same *k*_p_ as in SDS micelles [35]), we calculated *k*_inh_ for PMHC and for **NB-2**. The obtained kinetic parameters are listed in Table 1. Derivative **NB-1** (without phenol moieties) showed a slight retarding effect on the studied reaction, (see line 3 in Figure 5), but its activity was not sufficient to effectively break the peroxidation chain and no induction period was observed.

Compound **NB-2** has a noticeable effect on the rate of methyl linoleate peroxidation, decreasing it considerably, from *R*_ox_ = (4.3 ± 0.3) × 10^−7^ Ms^−1^ for spontaneous peroxidation to *R*_inh_ = (0.9 ± 0.1) × 10^−7^ Ms^−1^ for the sample containing 1 µM of **NB-2** (Figure 5), with the induction period τ_ind_ = 20.2 ± 0.8 min (average of 6 measurements). Based on eq. 7, we calculated the bimolecular rate constant for reaction of **NB-2** with peroxyl radicals, *k*_inh_ = 1000 ± 100 Ms^−1^, as it would be expected for a moderate antioxidant. In this case, we regarded the reactivity of **NB-2** being one order of magnitude smaller than tocopherols analogue (PMHC) as an advantage because the compound will perform its reporting role when other, more reactive antioxidants are exhausted in the system. Finally, from rearranged equation 6, with *R*_i_, [**NB-2**]_0_ and τ (in seconds) taken from Table 1, the stoichiometric coefficient *n* = 5.3 was calculated for **NB-2**. This parameter exceeds the predicted number of four radicals scavenged by two phenolic moieties, however, the presence of a double C=C bond conjugated with an aryl ring provides additional susceptibility to trap more radicals than could be expected for simple phenols.

### 3.3. UV-vis and Spectrofluorimetric Study of the Reaction of **NB-2** with Peroxyl Radicals in Methanol

**Figure 6:**
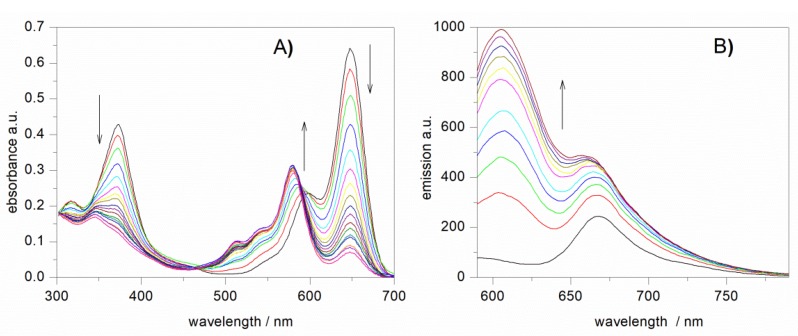
(**A**) absorption spectra of solution of 15 μM **NB-2** and 5 mM AIBN in methanol. Sample (optical path 10 mm) was periodically exposed to 5 sec. irradiation with 365 nm UV High Power LED; then, after 1 min, the spectrum was taken and the process was repeated for the same sample. (**B**) Emission spectra recorded with the same methodology of irradiation (λ_ex_ = 575 nm, slit = 5 and 5 nm, and gain = high).

With the knowledge that **NB-2** reacts with the peroxyl radicals mediating the peroxidation chain in a water/lipid biphasic system, we investigated whether this radical trapping molecule could report on the presence of peroxyl radicals. Figure 6 presents the results of several experiments monitoring the photochemical response of **NB-2** to peroxyl radicals generated from AIBN. At room temperature, during the photodissociation of AIBN in the liquid phase alkyl radicals are being produced with a moderately high quantum yield of ca. 0.44 (in benzene) [36], Reaction (8).

RN=NR+ hѵ → [RN=NR]* → [R• + R•] + N_2_. (8)

In an oxygen saturated solvent, once the R• radicals escape from the solvent cage (the cage effect), they immediately react with O_2_ producing peroxyl radicals (Reaction (2), with *k*_2_ ~10^9^ M^−1^·s^−1^). In our experiment, a sample of 5 mM AIBN in methanol containing 15 μM **NB-2** was periodically exposed to 365 nm UV light (5 s), and after 60 s, the absorbance and emission spectra were recorded (separate series of experiments). The obtained UV-vis spectra are presented in Figure 6A, demonstrating that oxidation (H atom abstraction from phenolic moieties, **NB-2** → **NB-2**_ox_) is accompanied by a ten-fold decrease of the absorbance at λ = 647 nm, a three-fold decrease at 373 nm and a two-fold increase of the absorbance at λ_max_ at 575 nm. Consequently, we decided to use 575 nm as the excitation wavelength for **NB-2**_ox_. Figure 6B presents the emission spectra a sample of **NB-2** reacting with peroxyl radicals (separate series but the same concentrations and conditions). As the oxidation of **NB-2** progresses, an intensive emission for **NB-2**_ox_ appears with a maximum at λ = 605–615 nm. The intensity of the initial red fluorescence at 674 nm originating from parent molecule (**NB-2**) increases much slower and overlaps with the aforementioned band at 605–615 nm. Emission intensity measured at 612 nm undergoes a ca. 14-fold enhancement, suggesting that **NB-2** is a promising candidate for a fluorescent probe for peroxyl radicals.

In the next series of experiments, the radicals were generated by thermolysis of AIBN in methanol. The quartz cell with the sample was placed in a thermostated pocket of the spectrofluorometer and emission at 612 nm was monitored on-line within 120 min. Upon thermolysis, the same primary alkyl radicals were produced (Reaction (9)) as during the photolysis of AIBN.

AIBN→ 2*f* R• + N_2_ (0 < *f* < 1)  ; *k*_d_. (9)

In an air-saturated solution, R• were immediately converted into peroxyl radicals (Reaction (2)), thus, peroxyl radicals were generated with a constant rate *R*_g_ = 2 *f k*_d_ [AIBN] and under 5 mM initial concentration of AIBN with *k*_d_ = 0.28 × 10^−6^ s^−1^ at 37 °C and 2.2 × 10^−6^ s^−1^ at 50 °C in benzene [37] with the assumption that *f*~0.5 [38] the values of *R*_g_ are 1.5 nMs^−^^1^ at 37 °C and 11 nMs^−^^1^ at 50 °C. This first value is comparable to *R*_i_, which was determined during peroxidation of LinMe in the micellar system in Section 3.2.

**Figure 7:**
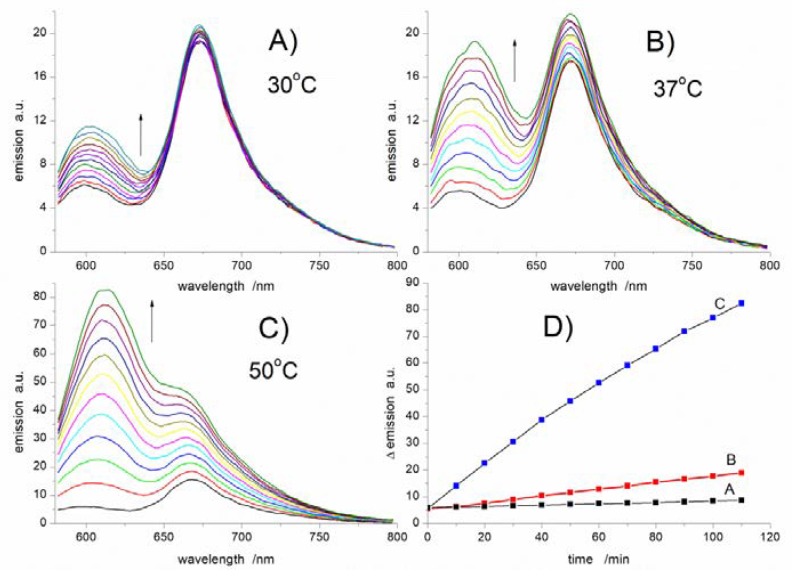
(**A**–**C**): Fluorescence spectra recorded for 15 μM **NB-2** and 5 mM AIBN in methanol at 30, 37, and 50 °C (upon excitation at λex = 575 nm). (**D**): Comparison of intensity/time profiles for the emission at 612 nm, read from the same data, as shown in (**A**–**C**).

The emission spectra presented in Figure 7A–C show that the progress of oxidation (H atom abstraction from phenolic hydroxyl groups) is manifested by the increased emission at 612 nm. The slopes of the lines in panel D can be considered as representations of the rates of oxidation of **NB-2**. The comparison of those slopes gives relative rates of oxidation 1:4:23 for the processes carried out at 30, 37, and 50 °C, respectively, which is in good agreement with the accessible rate constants, *k*_d_, for thermolysis of AIBN (Reaction (9)) and with *k*_d_ calculated from the Arrhenius equation with *E*_a_ = 128.9 kJ/mol and log*A* = 15.19 s^−1^ [38].

**Scheme 2:**
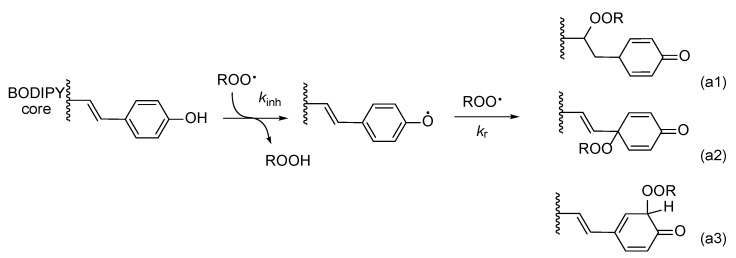
Reaction the hydroxycinnamyl residue (single one is presented for simplicity) with two peroxyl radicals ROO• and the formation of three isomeric products of recombination.

In both kinds of experiments carried out in methanol, with peroxyl radicals generated by photolysis and thermolysis of AIBN, we observed a maximal 14-fold fluorescence enhancement. Such enhancement cannot be assigned to processes other than the reaction with peroxyl radicals (we exclude the reaction with molecular oxygen even during prolonged time, *vide supra*, and see Figure S6). Therefore, we assume that the oxidized form of **NB-2** is responsible for emission at 605–612 nm and 674 nm. Scheme 2 presents a proposed mechanism of a two-step oxidation of one receptor segment of **NB-2**: H atom abstraction from the phenolic hydroxy group by a peroxyl radical and a subsequent recombination of the formed phenoxyl radical with another peroxyl. This proposed mechanism is in accordance with the standard mechanism of reaction of peroxyl radicals with derivatives of hydroxycinnamic acids, such as p-coumaric, caffeic, ferulic, and sinapic acids [39]. Since recombination of phenoxyl radical with ROO• is very fast (for *para*-substituted phenols *k*_r_ > 10^8^ M^−1^·s^−1^ [36]), the first reaction, H atom abstraction, with *k*_inh_ = 10^3^ M^−1^·s^−1^ will be the rate determining step. Each hydroxycinnamyl residue can react with two peroxyl radicals, giving *n* = 4, which is smaller than the experimentally determined *n* > 5 (Section 3.2). This effect can be explained by the possible addition of the next ROO• to double the C=C bond in structures a2 and a3.

### 3.4. UV-vis and Spectrofluorimetric Study of the Reaction of **NB-2** with Peroxyl Radicals in Micelles

**Figure 8:**
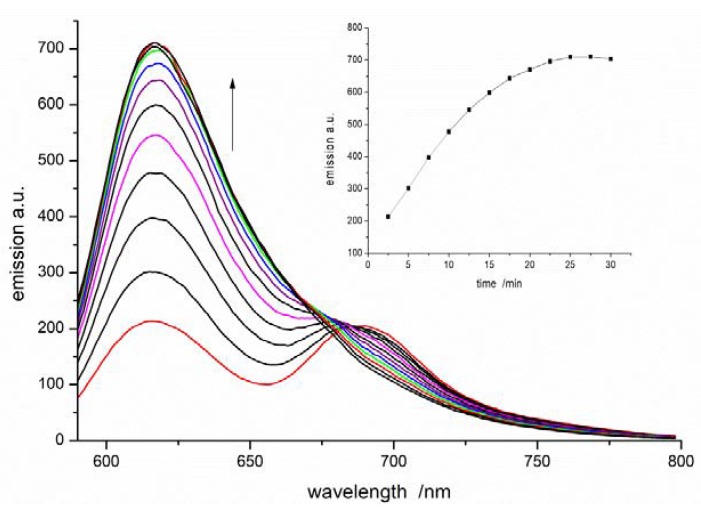
Emission spectra of 10 μM **NB-2** dissolved in 8 mM Triton X-100 micelles containing 2.74 mM methyl linoleate and undergoing peroxidation initiated with 10 mM ABAP at 37 °C and pH 7.4. Spectra were recorded every 2.5 min. Inset: Plot of emission intensity at 616 nm versus the time of peroxidation.

Using the excitation wavelength 575 nm (see previous section) for the visualization of oxidized **NB-2** is advantageous as for λ > 500 nm the signal distortion caused by autofluorescence in the biological and biology relevant systems is minimal. From this point of view, **NB-2** should be a highly suitable FP used for monitoring the peroxidation reactions however, some other environmental and microenvironmental factors can affect fluorescence and detectability, for example: pH changes, solvent polarity, background fluorescence, light scattering, and interactions of the fluorochrome with other fluorochromes present in the sample. Even though experiments with biological systems are beyond the scope of this preliminary report, we decided to check the behavior of **NB-2** in a micellar system the same as for the kinetic measurements described in Section 3.2, with ABAP producing the peroxyl radicals with the same rate of initiation, *R*_i_ = 4.3 nMs^−1^ (see Table 1). The emission spectra recorded for every 2.5 min during the peroxidation reaction are collected in Figure 8. The emission spectrum of **NB-2** in the micellar system before the oxidation started (Figure 8, red line, see also Figure S3) shows two bands with similar intensity: the first one with maximum absorbance at 674 nm and a second one at 616 nm. The cumulative plot presented in Figure 8 (inset) shows that the increase of emission recorded in the micellar system follows the same trend as in methanol (with AIBN). The observed 3.9-fold increase of fluorescence intensity during 20 min of reaction is lower than the analogous one in methanol, but is comparable to the 4-fold increase in emission observed by Cosa for his probe B-TOH (Figure 3) tested in phospholipid DMPC vesicles at pH 7.4 with the radicals generated with ABAP [25]. After 25 min of peroxidation, a slow decrease in emission (Figure 8, inset) can be noticed as an effect of radical-mediated BODIPY degradation at further stages of peroxidation [40].

We also monitored the changes in absorbance during the prolonged oxidation (3 h) carried out in the micellar system at pH 4.0 and 7.0. UV-vis spectra for these measurements recorded every 15 min during peroxidation are presented in the Supplementary Material (Figure S7 for stability test without peroxidation, and Figures S8 and S9 for peroxidation at pH 7 and pH 4). The observed effects are the same as for the reactions carried out in methanol, i.e., a slow decrease of intensity of broad bands with λ_max_ at 373 and 660 nm and parallel build-up of the bands at 587 and 513 nm. 

## 4. Conclusions

We designed and prepared a fluorescent probe **NB-2** and described its use for the detection of peroxyl radicals. This probe is composed of two receptor segments (4-hydroxycinnamyl moieties) sensitive towards peroxyl radicals that are conjugated with a fluorescent reporter, dipyrrometheneboron difluoride (BODIPY), whose emission changes depend on the oxidation state of the receptors.

According to the oxygen intake measurements performed with a Clark-type oxygen electrode, **NB-2** behaves like a moderate chain-breaking antioxidant during the peroxidation of methyl linoleate (LinMe) in Triton X-100 at pH 7, initiated with a water soluble azo-initiator, ABAP, at 37 °C. In this system, the rate constant for a reaction with peroxyl radicals (*k*_inh_) is 1000 ± 100 M^−1^·s^−1^, being one order of magnitude smaller than *k*_inh_ for reaction of PMHC (an analogue of α-tocopherol). The stoichiometric coefficient, *n,* determined for **NB-2** in this system, is above five, indicating that peroxyl radicals are trapped not only by phenolic moieties (which would result in *n* = 4), but also by other structural parts of **NB-2**.

The reporter segment of **NB-2** absorbs and emits light in the visible region of the spectrum (λ > 500 nm). The whole molecule shows thermal stability (up to 3 h in methanol saturated with oxygen) and chemical stability upon 30 min exposition to peroxyl radicals. UV-vis and spectrofluorimetric experiments demonstrated that **NB-2** yields a highly fluorescent product upon scavenging peroxyl radicals in a homogeneous solution (methanol), with AIBN used as a source of radicals generated by photolysis or thermolysis, and in a micellar system (LinMe/Triton X-100, pH 7), with ABAP used as a thermal source of peroxyl radicals. The emission enhancement upon such exposition to peroxyls is 14-fold in methanol and 4-fold in the micelles.

The preliminary results presented in this report show that **NB-2** is a promising novel lipophilic fluorescent “switch on” probe. Detailed photochemical characteristics, studies of selectivity and interactions with other antioxidants, measurements carried out in biologically relevant systems, and possible applications of **NB-2** will be presented in a full paper.

## Figures and Tables

**Table 1:** The lengths of induction periods, τ, the rates of initiation, *R*_i_, the slow-down factor (*R*_ox_*/R*_inh_, the ratio of the rate of non-inhibited process to the rate of inhibited process), and the inhibition rate constants, *k*_inh_, calculated for autoxidation of 2.73 mM LinMe dispersed in 8 mM Triton X-100 micelles in the presence of 1 µM PMHC, or **NB-1,** or **NB-2**. The experiments were performed at 37 °C and pH 7.0. Peroxidation was initiated by 10 mM ABAP. Each measurement was run 4–6 times (see Table S1). Values are expressed as the mean ± standard deviation (SD).

Experimental System	τ/min	*R*_i_*^a^*/nMs^−1^	*R*_inh_/nMs^−1^	*R*_ox_/*R*_inh_*^b^*	10^−3^ × *k*_inh_/M^−1^·s^−1^
PMHC	6.0 ± 0.6	4.3 ± 0.3	37 ± 13	11.6	12.1 ± 3.0
**NB-1**	- *^c^*	4.3 ± 0.3	220 ± 15 *^c^*	2.0	-
**NB-2**	20.2 ± 0.8	4.4 ± 0.4	90 ± 9	4.8	1.0 ± 0.1

*^a^* For *R*_i_ determination, see Equation (6). *^b^* The (*R*_ox_*/R*_inh_) ratio informs how many times the inhibited oxidation is slower than spontaneous (non-inhibited) process, with *R*_ox_ = (4.3 ± 0.3) × 10^−7^ Ms^−1^. It can be also considered as the ratio of the kinetic chain length (the number of propagating cycles) of the inhibited to non-inhibited process. *^c^* For this system, the inhibition period was not detected (see curve 3 in Figure 5) and the rate of the retarded process is listed.

## Supplementary Materials

The following are available online at https://www.mdpi.com/2076-3921/9/1/88/s1, Figures S1–S3, respectively: ^1^H, ^13^C, ^19^F NMR spectra of **NB-2**, Figure S4: HRMS (High Resolution MS) spectrum of NB-2, Figure S5: UV Irradiation Chamber, Figure S6: Absorption spectra of **NB-2** in methanol at 37 °C during 180 min, Figure S7: Stability of **NB-2** in micellar system at 37 °C and pH 7.4, Figure S8: UV-vis spectra recorded during peroxidation of methyl linoleate in Triton X-100 micelles containing **NB-2** at 37 °C and pH 7.0, Figure S9: UV-vis spectra recorded during peroxidation of 2.74 mM methyl linoleate in Triton X-100 micelles containing **NB-2** at 37 °C and pH 4.0, Table S1: The lengths of induction periods, τ_ind_, the rates of initiation, *R*_i_, kinetic chain length, *v*_ox_, *v*_inh_, *v*_ox1_ and the inhibition rate constants, *k*_inh_, determined for peroxidation of LinMe/Triton X-100 micelles inhibited by of PMHC, **NB-1** or **NB-2**.

## Abbreviations

ABAP	2,2′-azobis(2-amidinopropane)
AIBN	α,α′-azobisisobutyronitrile
BODIPY	dipyrrometheneboron difluoride
CBA	chain-breaking antioxidant
DCM	dichloromethane
DDQ	2,3-Dichloro-5,6-dicyano-1,4-benzoquinone
DMPC	1,2-dimyristoyl-sn-glycero-3-phosphocholine
FP	fluorescent probe(s)
LinMe	methyl linoleate
LH	lipid molecule
PMHC	2,2,5,7,8-pentamethylchroman-6-ol
PUFA	polyunsaturated fatty acids
R^•^	alkyl radical
LOO^•^	peroxyl radical
ROS	Reactive Oxygen Species
TEA	triethylamine
TFA	trifluoroacetic acid
THF	tetrahydrofuran
TLC	thin-layer chromatoraphy
Triton X-100	polyethylene glycol p-(1,1,3,3-tetramethylbutyl)-phenyl ether
