# Supplementary material for: Hydroxycinnamyl Derived BODIPY as a Lipophilic Fluorescence Probe for Peroxyl Radicals
Source: Antioxidants (Basel). 2020 Jan 20;9(1):88. doi: 10.3390/antiox9010088 (PMC7022944; doi:10.3390/antiox9010088)
Supplement: Supplementary file 1 [file antioxidants-09-00088-s001.pdf]

Supplementary Material

Hydroxycinnamyl derived BODIPY as a Lipophilic Fluorescence Probe for Peroxyl Radicals

Jaroslaw Kusio[1,a], Kaja Sitkowska[1,2,a,*], Adrian Konopko[1,3] and Grzegorz Litwinienko[1,*]

[a] J. Kusio and K. Sitkowska contributed equally to this work
[1] Faculty of Chemistry, University of Warsaw, Pasteura 1, 02-093 Warsaw, Poland
[2] Centre for Systems Chemistry, Stratingh Institute for Chemistry, University of Groningen, Nijenborgh 4, 9747 AG Groningen, The Netherlands
[3] Nencki Institute of Experimental Biology, Polish Academy of Sciences, 3 Pasteur St, Warsaw, 02-093, Poland
* Correspondence: litwin@chem.uw.edu.pl (G.L.), kasitkowska@gmail.com (K.S.)

TABLE OF CONTENTS

Title		page
**Figure S1.**	^{1}H NMR spectrum of **NB-2**.	S2
**Figure S2.**	^{13}C NMR spectrum of **NB-2**.	S2
**Figure S3.**	^{19}F NMR spectrum of **NB-2**.	S3
**Figure S4.**	HRMS (ESI+) spectrum of **NB-2**.	S3
**Figure S5.**	UV Irradiation Chamber.	S4
**Figure S6.**	Absorption spectra of **NB-2** in methanol at 37°C during 180 minutes.	S4
**Figure S7.**	Stability of NB-2 in micellar system at 37°C and pH 7.4.	S4
**Figure S8.**	UV-Vis spectra recorded during peroxidation of methyl linoleate in Triton X-100 micelles containing **NB-2** at 37°C and pH 7.0.	S5
**Figure S9.**	UV-Vis spectra recorded during peroxidation of methyl linoleate in Triton X-100 micelles containing **NB-2** at 37°C and pH 4.0.	S5
**Table S1**.	The lengths of induction periods, τ_{ind}, the rates of initiation, R_i, kinetic chain length, ν_{ox}, ν_{inh}, ν_{ox1} and the inhibition rate constants, k_{inh}, determined for peroxidation of MeLin/Triton X-100 micelles inhibited by of PMHC /or **NB-1** /or **NB-2**.	S6

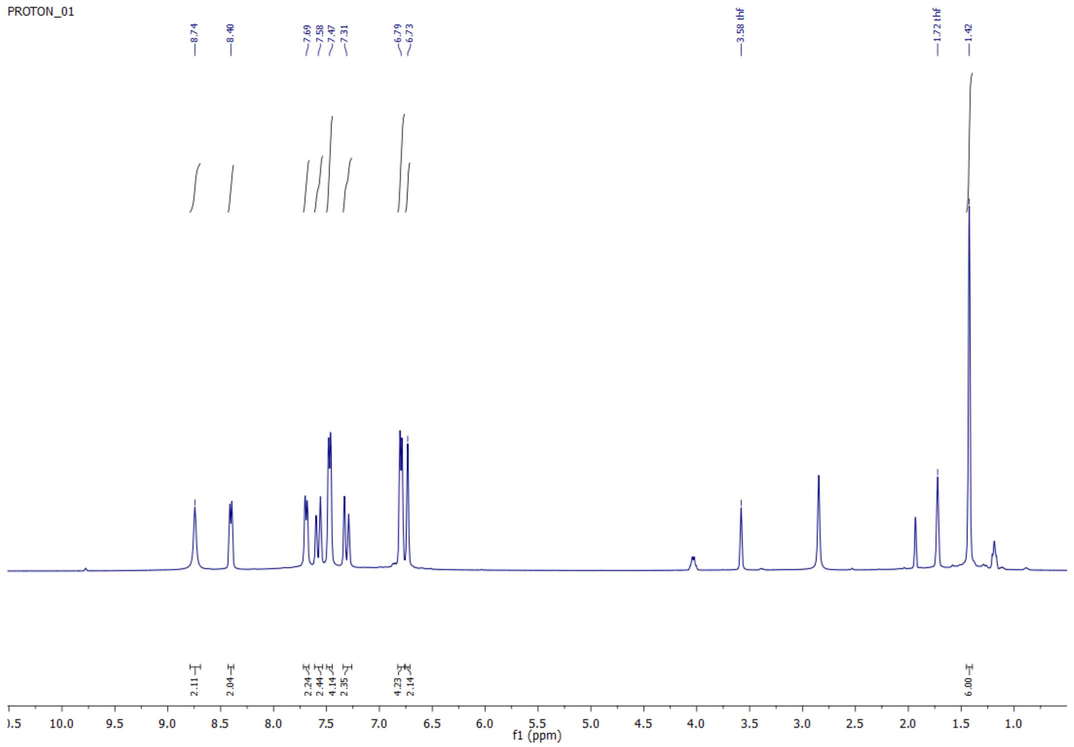

Figure S1. ^{1}H NMR spectrum of **NB-2** (400 MHz, THF-d_8) δ 1.42 (s, 6H), 6.73 (s, 2H), 6.79 (dq, J = 8.8, 2.1 Hz, 4H), 7.31 (dd, J = 16.3, 3.0 Hz, 2H), 7.43 – 7.50 (m, 4H), 7.57 (d, J = 16.3 Hz, 2H), 7.63 – 7.74 (m, 2H), 8.40 (dq, J = 8.8, 2.1 Hz, 2H), 8.74 (brs, 2H).

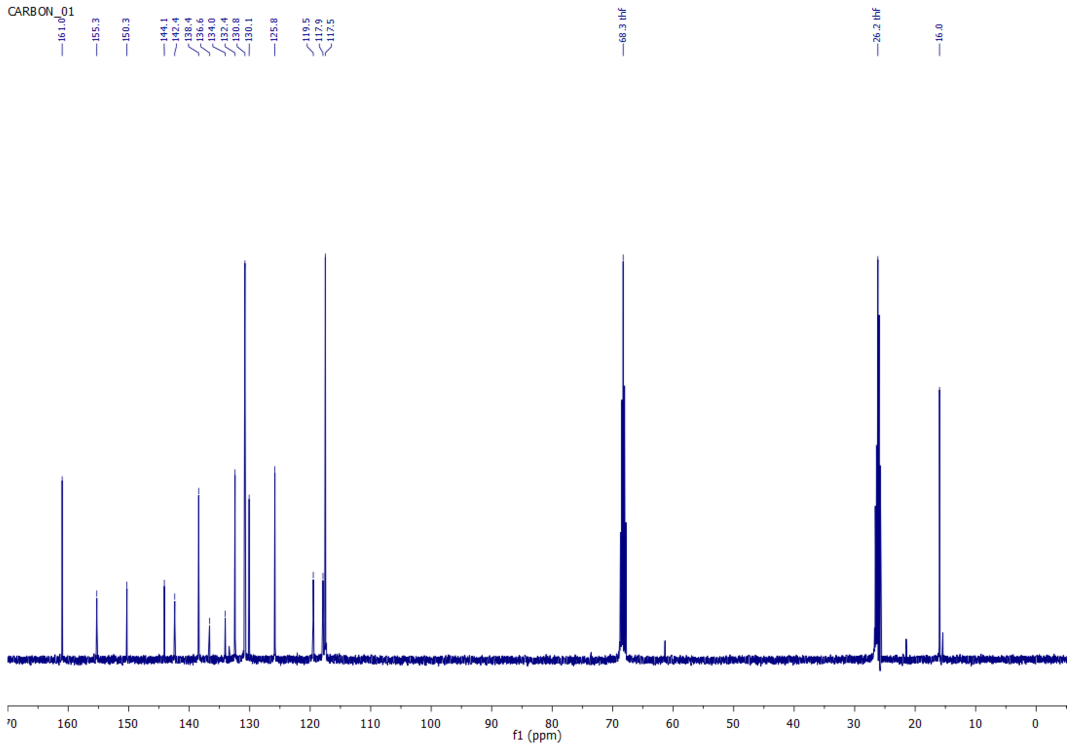

Figure S2. ^{13}C NMR spectrum of **NB-2** (101 MHz, THF-d_8) δ 16.0, 117.5, 117.9, 119.5, 125.8, 130.1, 130.8, 132.4, 134.1, 136.7, 138.4, 142.4, 144.1, 150.3, 155.3, 161.0.

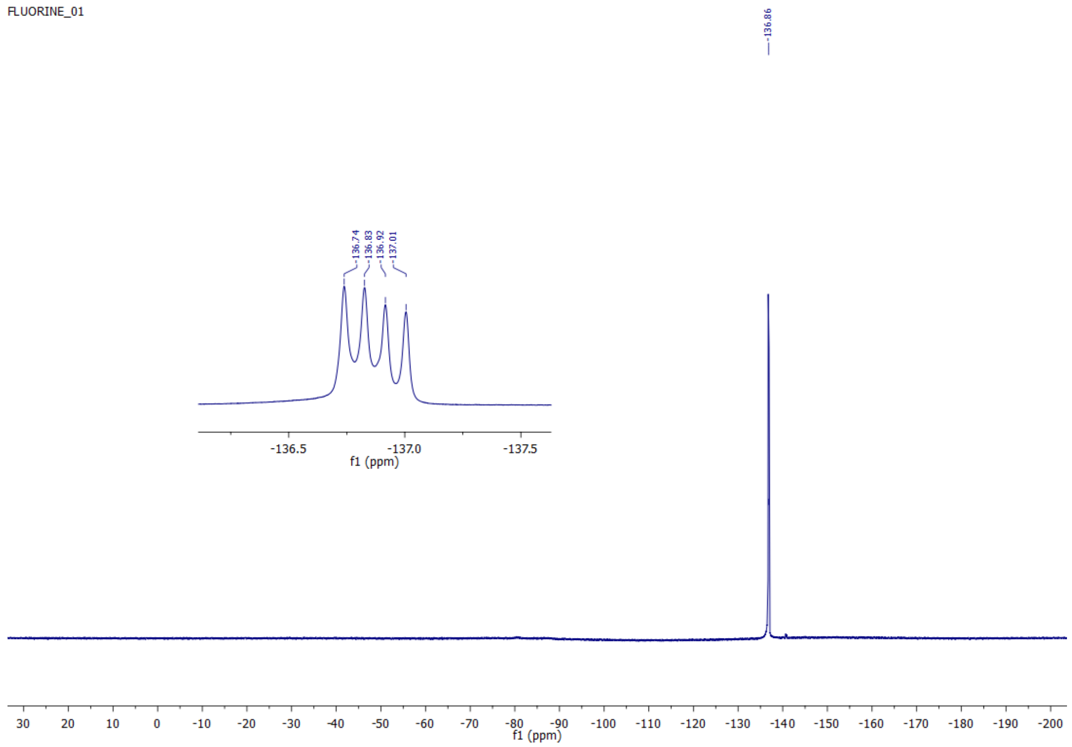

Figure S3. ^{19}F NMR spectrum of **NB-2** (376 MHz, THF-d_8) δ -136.86 (dd, J = 67.0, 33.1 Hz).

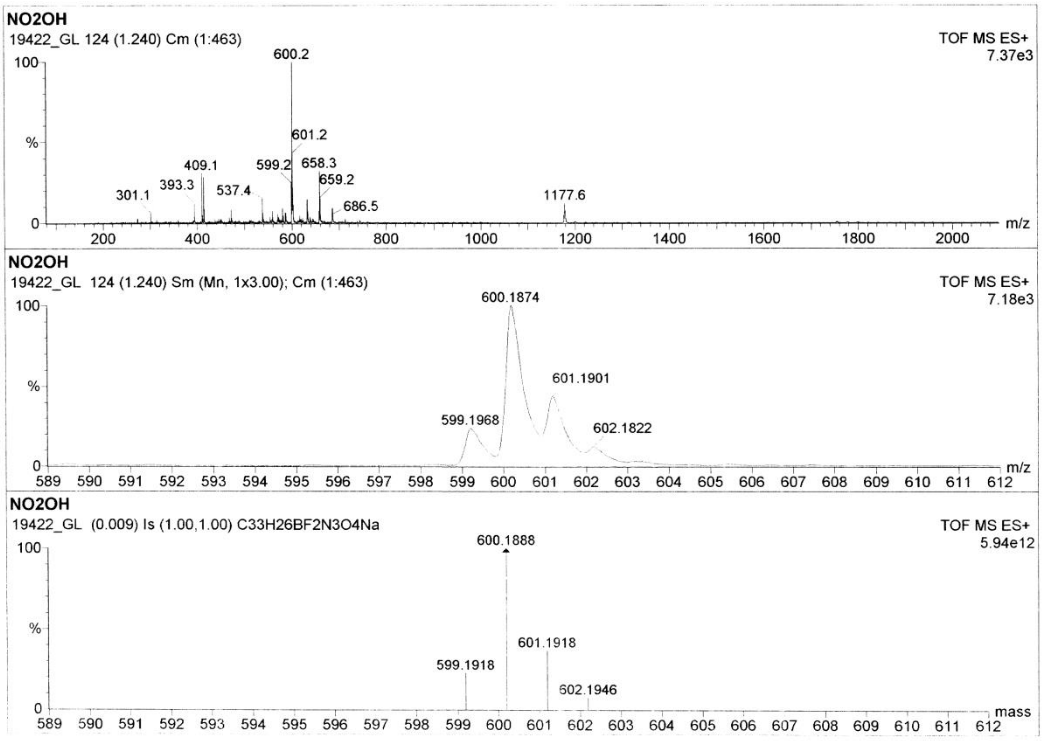

Figure S4. HRMS (ESI+) spectrum of **NB-2** calc. for $[M+Na]^+$ ($C_{33}H_{26}BF_2N_3O_4Na$): 600.1888, found: 600.1874.

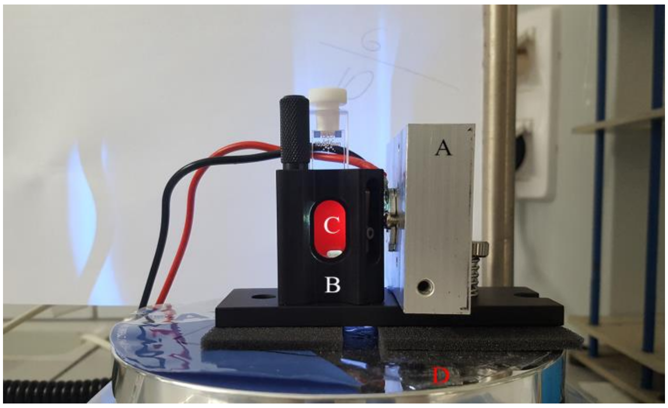

Figure S5. UV Irradiation Chamber: UV LED 365 nm with aluminium radiator (A), cuvette holder with in/out windows (B), quartz fluorescence cuvette filled with solution of compound (C), all elements standing on the magnetic stirrer plate (D).

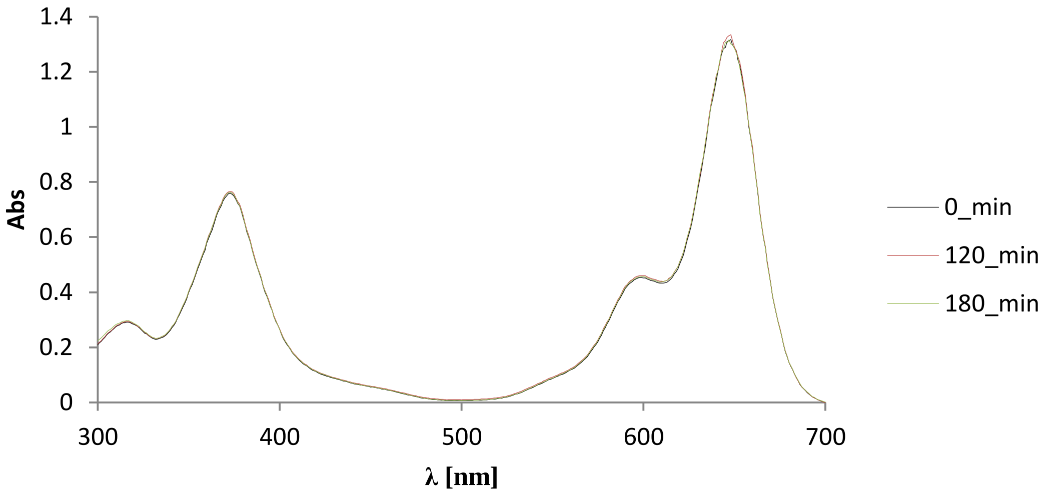

Figure S6. Absorption spectra of **NB-2** in (~20 μM) methanol at 37°C during 180 minutes.

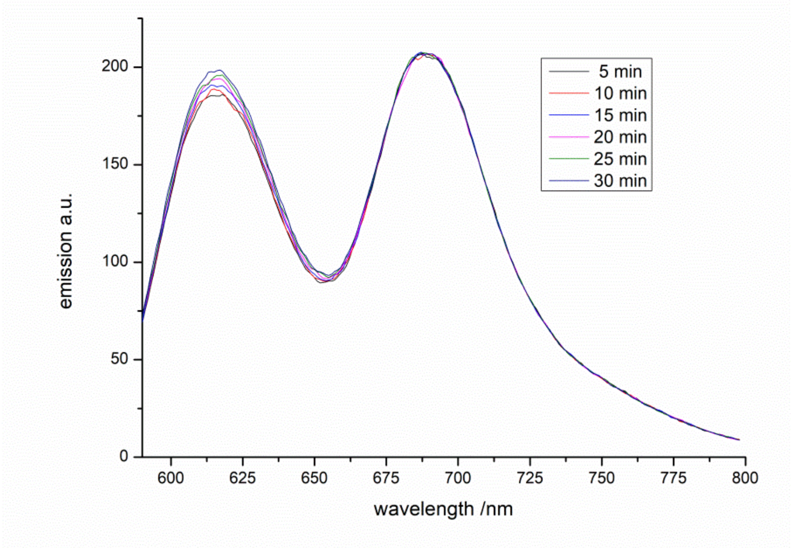

Figure S7. Stability of **NB-2** (10 μM) in micellar system (8 mM Triton X-100 micelles containing 2.74 mM methyl linoleate) at 37°C and pH 7.4 (phosphate buffer). Emission spectra recorded at 585-800 nm, λ_{ex} = 575 nm.

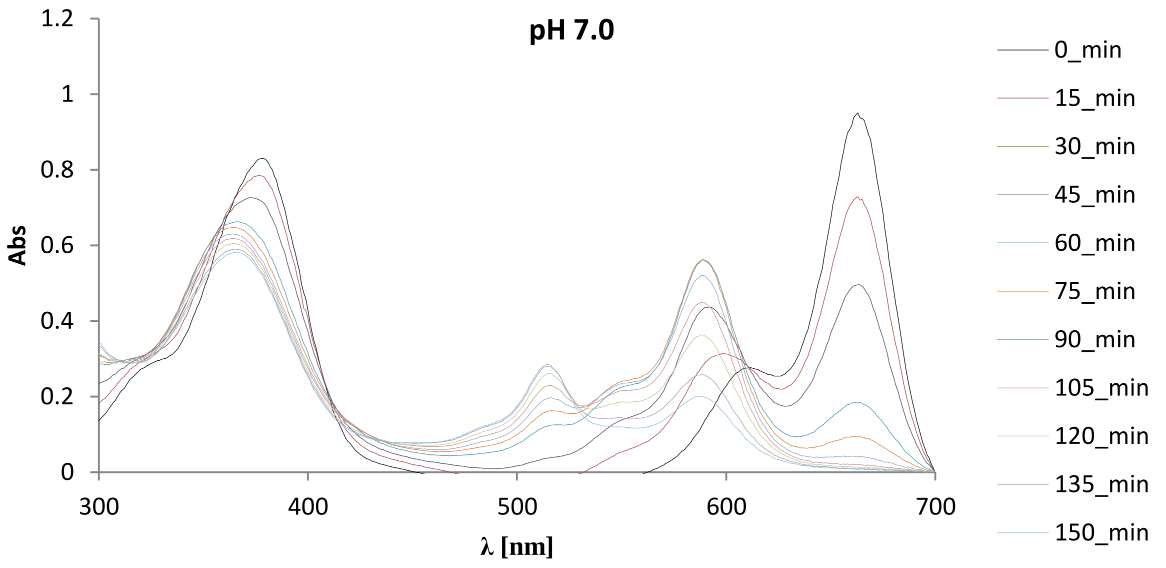

Figure S8. UV-Vis spectra recorded every 15 min. during peroxidation of 2.74 mM methyl linoleate in 8 mM Triton X-100 micelles containing 9.0 μM **NB-2** at 37°C and pH 7.0 (Tris buffer). Peroxidation was initiated with 25 mM ABAP.

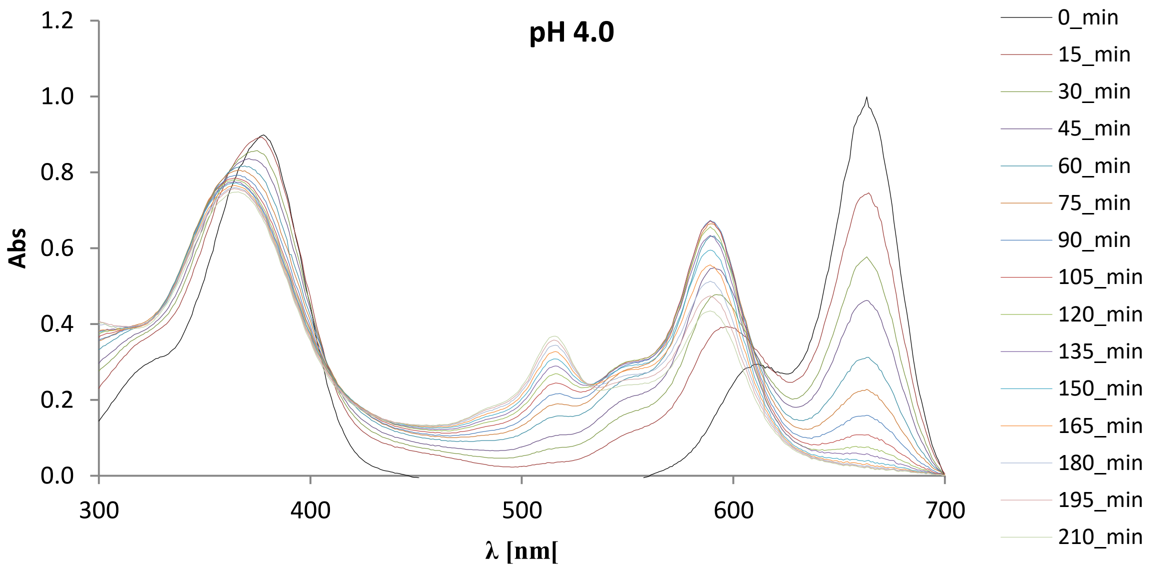

Figure S9. UV-vis spectra recorded every 15 minutes during peroxidation of 2.74 mM methyl linoleate in 8 mM Triton X-100 micelles containing 9.0 μM **NB-2** at 37°C and pH 4.0 (acetate buffer). Peroxidation was initiated with 25 mM ABAP.

Table S1.The lengths of induction periods, τ_{ind}, the rates of initiation, R_i, kinetic chain length, v_{ox}, v_{inh}, v_{ox1} and the inhibition rate constants, k_{inh}, determined for peroxidation of MeLin/Triton X-100 micelles inhibited by 1 μM PMHC /or **NB-1**/ or **NB-2**. Experiments were performed in 8 mM Triton X-100 micelles with 2.73 mM MeLin at 37°C, pH 7.0. Peroxidation was initiated by 10 mM BAP. All experiments were repeated 3-6 times. Values are expressed as the mean ± standard deviation (SD).

Compound	τ /min	R_i /nMs^{-1}	R_{inh} /nM^{-1}	$k_{inh} \times 10^{-3}$ /M^{-1}s^{-1}	$R_{ox} \times 10^7$ /M^{-1}	$R_{ox1} \times 10^7$ /M^{-1}	v_{ox}[a]	v_{inh}[a]	v_{ox1}[a]
PMHC	6.0±0.6	4.3	37±13	12.1±3.0	4.3±0.3	2.9±0.2	100	9	67
NB-1	- [b]	4.3	220±15 [b]	-	4.3±0.3	-	100	51	-
NB-2	20.2±0.8	4.3	90±9	1.0±0.1	4.3±0.3	1.8±0.1	100	21	42

[a] The kinetic chain length v is the number of peroxidation cycles triggered by one initiating radical. Here, for non-inhibited peroxidation, $v_{ox1}=R_{ox1}/R_i$. [b] For this system, the inhibition period was not detected (see curve 3 in Figure 5) and the rate of the retarded process is listed.
